# Development and evaluation of the first fertility preservation patient decision aid to support boys and young men with cancer: The Cancer, Fertility and Me for Boys and Young Men research protocol.

**DOI:** 10.1136/bmjopen-2025-104475

**Published:** 2025-08-22

**Authors:** Georgina Louise Jones, Danielle Sarah Musson, Nicola O’Donnell, Abigail Lewis, Maximilian Williamson, Dan Yeomanson, Allan Pacey, Sheila Lane, Anne-Mairead Folan, Brendan Gough, Bob Phillips, Carol Porteous, Richard Anderson, Rod Thomas Mitchell

**Affiliations:** 1 Psychology, School of Humanities and Social Sciences, Leeds Beckett University, Leeds, UK; 2 Centre for Reproductive Health, University of Edinburgh, Edinburgh, UK; 3 Queen Elizabeth University Hospital, Glasgow, UK; 4 Sheffield Children’s NHS Foundation, Sheffield, UK; 5 School of Medical Sciences, Faculty of Biology, Medicine and Health, University of Manchester, Manchester, UK; 6 Oxford University Hospitals NHS Trust, Oxford, UK; 7 Centre for Reviews and Dissemination, University of York, York, UK; 8 The Hull York Medical School, University of York, York, UK; 9 Edinburgh Clinical Research Facility, University of Edinburgh, Edinburgh, UK

**Keywords:** ONCOLOGY, Paediatric oncology, Male infertility, REPRODUCTIVE MEDICINE, Decision Making

## Abstract

**Abstract:**

**Introduction:**

Many cancer treatments can result in reduced fertility, impacting survivors’ opportunities for biological parenthood. Fertility preservation (FP) methods for boys and young men, such as cryopreservation of testicular tissue or sperm, offer hope but are currently underused among young male patients with cancer. Despite guidelines recommending early discussion of fertility implications, many newly diagnosed males do not receive FP counselling or referral to fertility services. Male cancer survivors face a higher likelihood of infertility than their peers, yet focused FP decision-making support is lacking. This study aims to address this gap by developing and evaluating the first dedicated patient decision aid (PtDA) for boys and young male patients with cancer aged 11–25 years old, to help them make informed FP decisions before receiving cancer treatment.

**Methods and analysis:**

The current study follows a multistage process: developing the PtDA, alpha testing for acceptability with former patients, parents and healthcare professionals, and beta testing in clinical settings to ensure effective integration into routine care. Using a combination of interviews and questionnaire data, this research will assess the PtDA’s acceptability and impact on decision-making.

**Ethics and dissemination:**

This study has been prospectively registered on the Research Registry (10273). Ethics approval has been obtained from Leeds Beckett University and the National Health Service/Health Research Authority before undertaking data collection. The final resource will be disseminated widely and made freely available online via our dedicated Cancer, Fertility and Me website, for use in clinical and research practice.

**Strengths and limitations of this study**

- This research will build on our established Cancer, Fertility and Me decision aid programme of work that was originally designed for females.
- The research will provide evidence of its acceptability and utility to boys and young men, parents and healthcare professionals in usual practice across cancer and fertility care pathways.
- This research will identify possible issues for implementation in routine clinical care.
- This research will not provide evidence of the patient decision aid (PtDA)’s effectiveness on healthcare outcomes. Instead, our findings will inform future studies designed to evaluate the efficacy of the PtDA on health outcomes for boys and young men.

## Introduction

 One of the most distressing late effects of cancer treatment is infertility, often denying patients with cancer the opportunity to have their own biological children.^1 2^ Loss of fertility is dependent on several factors, including the cancer diagnosis and the specific treatment regimen (chemotherapy/radiotherapy) that the individual receives.^3^ Fertility preservation (FP) treatments (eg, cryopreservation of sperm or testicular tissue) provide much-needed hope for young men and their families. UK guidelines, such as those produced by the National Institute for Health and Care Excellence, state that cancer teams should discuss the impact of cancer treatment on future fertility at diagnosis, enabling patients to consider their options.^4^,^6^ Nonetheless, despite the increase in available FP options and their increasing efficacy, evidence suggests that many patients with cancer are either not considered, not referred or inappropriately referred for FP.^7^,^10^

Existing research suggests that male childhood cancer survivors are half as likely to father a child as their siblings.^11^ Sperm cryopreservation is a well-established method of FP for pubertal and adult patients, while testicular tissue freezing is increasingly being offered to prepubertal boys. These methods offer hope for men and their future partners to undergo future IVF or related fertility treatments.^12^ However, although many male patients are childless at cancer diagnosis, evidence suggests that only around 50% of young adult men are offered sperm freezing,^13^ despite this being a long-established, non-invasive, quick and low-cost process.^14^ Males often describe feeling inadequately supported in making FP decisions and report a lack of information to support FP decision-making.^14^ In addition, decisions about FP depend on their health at diagnosis, information provided and interactions with clinical care. It is also recognised that extra care should be taken in counselling younger males who may have given little or no consideration to future parenting.^15^

To date, research in the area of cancer and FP has focused primarily on female patients with cancer, but we know that many male patients need support in making decisions to have children.^13^ Men who experience infertility report significant impacts on well-being, personal identity and relationships with partners, while also highlighting a lack of support from healthcare professionals (HCPs).^16 17^ However, resources which provide information and support FP decision-making in males are currently lacking. A UK study of 100 oncologists found that 87% expressed a need for more FP information; however, only 38% reported routinely providing patients with written information, and one-third did not usually refer patients with fertility questions to a specialist service.^18^ In another survey, only 73% of oncologists reported routine discussions around sperm cryopreservation. Most (92%) would only refer men aged 20–40 years and were unaware that sperm cryopreservation does not delay the start of cancer treatment.^19^

One type of psychological intervention which can support patients with cancer and HCPs with FP decision-making is patient decision aids (PtDAs) (otherwise known as decision support interventions). Decision aids help patients, alongside their HCPs, to make deliberate, personalised choices regarding their healthcare.^20^ These are particularly important for decisions where there is some uncertainty about a specific course of action, as is often the case for this population. A recent systematic review has demonstrated the positive impact of PtDAs: patients gain knowledge, greater understanding of probabilities and increased confidence in their decisions.^21^ Currently in the UK, only teenage and adult women have access to an FP decision aid (developed by members of this team); there is no equivalent provision for boys and young men.^22^

In 2009, an educational resource, ‘Banking of Fatherhood’, was developed to support FP decision-making, but focused only on sperm banking among males aged 14–45 years.^23^ Recently, a paper was published by a team in Switzerland exploring the FP needs and experiences of male patients with cancer. Although the mean sample age was 32.9 years, most men felt that further resources to support FP decision-making (including information about sexuality, experiences from other patients, consequences for partners and virility) were needed.^24^ To the best of our knowledge, there is currently no such resource tailored to boys and young men aged 11–25 years, which is an inequality this study aims to address.^25^ The need for this dedicated resource has also been confirmed by our National Institute for Health and Care Research-funded public consultation with male patients with cancer in February 2022, with men indicating a strong preference for targeted information aimed at facilitating FP decision-making. Our project will address this need by developing the first dedicated PtDA for young male patients with cancer aged 11–25 years old, to enable them to consider their FP options before starting cancer treatment.

### Aims and objectives

The aim of this study is to develop and assess the acceptability of the first dedicated PtDA designed to support boys and young male patients with cancer with the FP treatment decision before starting cancer treatment. The need, feasibility and objectives for the study have been outlined above. The resource will be developed and evaluated in four stages in line with best practice methodological recommendations, including the International Patient Decision Aids Standards (IPDAS) and other decision science guidelines.^26^ ^27^

Our objectives are to:

1. Develop a PtDA to support young male patients with cancer to make FP choices, following a recent cancer diagnosis.
2. Alpha test the acceptability of the PtDA with boys and young men, their parent/carers (where relevant) and oncology and fertility service staff. This will include an assessment of its usefulness in planning care and making decisions between treatment options, the likelihood of use and barriers to use in practice.
3. Beta/field test the PtDA to evaluate its acceptability as part of routine care with boys and young men, their parents/carers (where relevant), and oncology and fertility service staff.
4. Produce the final version of the PtDA and disseminate widely. The resource will be made publicly available for free on the Cancer, Fertility and Me (CFM) PtDA website, alongside the female equivalent(s).

## Methods and analysis

### Design

**Figure 1:**
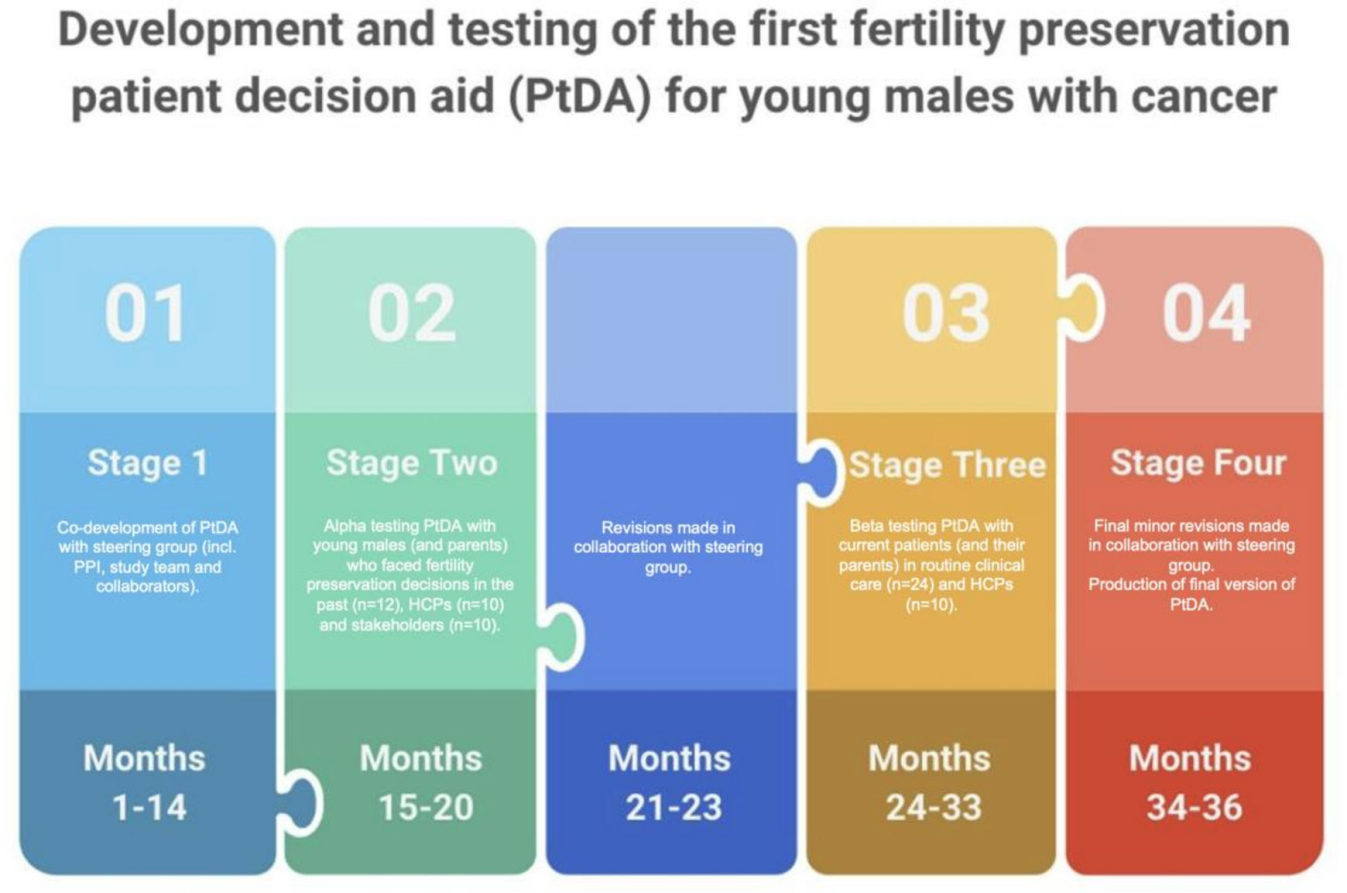
A diagrammatical overview of the four stages of the PtDA development and evaluation process: development of the PtDA (stage 1), alpha testing the PtDA (stage 2), beta testing the PtDA (stage 3) and producing and disseminating the resource and study findings (stage 4). HCPs = healthcare professionals; PPI = patient and public involvement.

The CFM PtDA for boys and young men will be developed over 3 years using systematic and evidence-based methods. A prospective, observational study using interview and questionnaire methodology will be used to evaluate the PtDA. This will be informed by the Medical Research Council Guidance for Developing Complex Interventions.^28^ The research plan includes four stages: development of the PtDA (stage 1), alpha testing the PtDA (stage 2), beta testing the PtDA (stage 3) and producing and disseminating the resource and study findings (stage 4). These are described below and can be seen in figure 1.

### Patient and public involvement (PPI)

Consultations with our PPI group informed the development of this study protocol. With support from the National Cancer Research Institute (NCRI), a PPI event was organised. The NCRI prepared and circulated an advertisement on social media, targeting relevant teenage and young adult cancer organisations. Five young men from the Teenage Cancer Trust took part and shared their views on study conceptualisation and design, including prioritisation of research questions and choice of data collection methods and recruitment strategies. Two members of staff from the NCRI, the two co-leads and an illustrator were also present so that PPI discussions could be captured visually. Following the meeting, one PPI member subsequently became a co-applicant on this study. PPI members will be involved in all phases of this project and will support the dissemination of research findings.

### Stage 1: development of the PtDA (months 1–14)

#### Conceptual framework

A prescriptive framework will guide the development of the PtDA, that is, it will help boys and young men make deliberate decisions, evaluating each option and its consequences, alongside their values. The decision theory-centred Ottawa Decision Support Framework has been chosen as it is particularly suitable when the decision in question (ie, to preserve fertility or not) is preference sensitive.^29 30^ The nature of PtDAs can vary from brief tools for use in synchronous encounters (face-to-face or mediated by other means); or more extensive tools (booklet, video or websites) that HCPs recommend patients to use, either before and/or during clinical encounters.^31^ Based on the existing evidence and PPI feedback, our aim is to develop an extensive tool that oncologists and nursing teams will offer to their young male patients with cancer.

#### Developing the PtDA prototype

The active components of the PtDA will be informed by our: (1) current systematic review (manuscript in preparation) which has identified boys and young men’s values and experiences of FP decision-making in the context of cancer; (2) consideration of relevant clinical evidence to identify levels of risk associated with FP treatments for this age group; (3) consultations with a wider steering group which will comprise additional PPI members and other clinical, academic and key stakeholders (eg, charities); (4) existing CFM FP PtDAs for teenage and adult women^32^; (5) consideration of guidance for producing health information for younger people and (6) engagement with web-design and graphic design teams. It is likely that the content of the PtDA will cover the following:

- Explicit information about, and description of, the decision to be made (ie, helping boys and young men with cancer to make decisions about FP treatment before starting cancer therapy).
- Descriptions of the health problems (ie, cancer, fertility and the male reproductive system, potential impact of cancer treatments on male fertility).
- Information, in visual, textual, numerical (%) and table formats, to describe treatment options (including benefits/harm/consequences), which also include avoiding or postponing intervention (ie, no FP).
- Tailored information for each of the following: relevant patient groups ensuring respect for diversity; features of the intervention, including where the FP treatment option may be considered a newer treatment method; and implications for achieving fertility and chances of cancer recurrence.
- Guidance for communication and deliberation about the FP decision with HCPs and significant others (eg, via suggested questions to use, spaces to write and list their thoughts about each option, exercises to think about the importance of referral and help for young males (and any parent/carers) to clarify their own values, potentially including other patient stories).
- Other important information related to the FP decision, for example, around accessing and using stored tissue/sperm.
- Other components (ie, information about useful contacts, sources of evidence, the team).
- A one-page summary table, for potential use as an option grid offering structured comparison of the different options, allowing the user a method to make a comprehensive evaluation of their choices.

#### Format

Following feedback from our PPI group, we will make the PtDA available in paper and electronic formats. Initially, we plan to make a PDF version of the resource accessible via our existing CFM PtDA website (www.cancerfertilityandme.org), ensuring that boys and young men have their own dedicated landing page. So far, there have been over 40 000 visitors to this website.

### Stage 2: alpha testing the PtDA (months 15–23)

Stage 2 aims to assess whether the prototype version of the PtDA is acceptable, comprehensible and usable to past patients, parents and HCPs (ie, to determine if it has face validity).^27^

Relevant stakeholders will be asked to review the resource. All young male volunteers will be purposively selected to include different ages, cancer types, ethnic backgrounds and FP treatment choices made. We will also seek to recruit participants with language difficulties such as dyslexia to ensure that the text is suitable for these patient groups. In particular, we will recruit three groups:

- Group 1: 12 past patients (ie, boys and young men aged 11–25 years old who previously faced the FP treatment decision in the past and are now at least 12 months post diagnosis), recruited from hospital sites across Leeds, London, Edinburgh and Sheffield. Additional sites may be approached to participate in our study if recruitment appears more challenging than anticipated. In stage 2, these sites will operate as patient identification centres (PICs). Clinical teams at the respective hospital sites will identify potentially eligible patients from their existing clinical databases. Eligible participants (or their parents/carers if aged 11–15 years) will complete consent to contact forms and, on receipt of these, the research team will supply the resource, participant information sheets and consent forms via their preferred method of contact. Depending on the wishes of the patients, their parents/carers may be invited to review the resource and provide feedback either alongside their child or during a separate interview. Adverts will also be posted on social media sites, instructing potential participants to contact the research team directly if they are interested in participating.
- Group 2: 10 HCPs who care for and manage boys and young men diagnosed with cancer will be invited to review the resource and provide feedback during a remote interview. HCPs will be identified through the UK-wide Children’s Cancer and Leukaemia Group (CCLG) who have provided support in principle for this initiative.
- Group 3: 10 key stakeholders including our NCRI panel, teenage cancer relevant organisations (eg, Lymphoma Action and the Teenage Cancer Trust) and non-cancer relevant organisations (eg, the Scottish Dyslexia Association, local secondary schools with LGBTQ+ groups) across Edinburgh, Sheffield and Leeds will be contacted to provide feedback on the resource. Potential stakeholders, identified through our professional networks, will be contacted directly.

#### Data collection

Participants will be asked to review the PtDA for clarity, comprehension and relevance. They will also be asked to complete a modified version of the QQ-10, a measure of face validity consisting of 10 items relating to their views on the new tool, which we have applied successfully in previous PtDA studies.^33^ Follow-up individual telephone interviews will be carried out to seek clarification and gain more in-depth information on their perception of the PtDA.

#### Analysis

The feedback will be recorded, digitised and transcribed. Domain scores will be calculated for the QQ-10 following the questionnaire’s scoring algorithms. Open-ended QQ-10 responses and interview transcripts will be analysed using a practical content analysis approach, identifying any changes required to the PtDA. A deductive analysis will be conducted, applying an analytical framework to determine the overall attractiveness (ie, does the PtDA content appeal to the target audience?), comprehension (ie, do the target audience understand the content?), self-efficacy (ie, do the target audience feel the message is appropriate for them?), cultural acceptability (ie, do the target audience perceive the message to be salient and acceptable?), persuasion (ie, does the PtDA support the target audience to take action?) and usability of the PtDA (ie, in what ways did the target audience use the resource?). An inductive analysis will be conducted simultaneously, adding any new codes which fall outside of our analytical framework where relevant. The analysis will be performed by team members with experience in conducting these approaches. Once the feedback has been collated, the PtDA will be revised prior to field testing in stage 3.

### Stage 3: beta testing the PtDA: pilot study (months 24–33)

Stage 3 will aim to evaluate whether the resulting PtDA is acceptable to current patients and their HCPs. To do this, we will administer the PtDA into the cancer care pathway across seven UK centres that provide FP for boys and young men with cancer.^27^

The sample will be opportunistic and identified, over a 6-month period, from a cohort of boys and young men referred to children and adult hospitals with a new diagnosis of cancer and at risk of losing their fertility due to treatment. Recruitment will take place at the Royal Infirmary, Edinburgh, and Western General Hospital (Edinburgh); Sheffield Children’s Hospital and Weston Park Hospital (Sheffield); St James’s Hospital and Leeds General Infirmary (Leeds) and University College London Hospital (London). In stage 3, all National Health Service (NHS) trusts will operate as research sites, except for NHS Lothian, which will remain a PIC site (following the recruitment methods outlined in stage 2). Epidemiology suggests that most of these individuals will have a diagnosis of leukaemia, lymphoma, brain tumours or other solid cancers. All boys and young men aged 11–25 years with a new diagnosis of cancer, facing gonadotoxic cancer treatment and considering FP will be eligible. FP is discussed with approximately 500 young males per year across these sites. Considering a 20% non-response rate, we could recruit approximately 166 boys and young men during a 5-month period. This should mean that our qualitative sample size of 24 young males (and possibly their parents/carers) is easily achievable across all sites; however, more sites might be approached to participate in our study if recruitment appears more challenging than anticipated. All eligible boys and young men (and their parents/carers) will be informed about the PtDA by their oncology team during the first consultation, where their cancer treatment options are being discussed. They will be provided with a link to the resource, along with the patient information sheet and interview consent form, by their HCP. This follows the ‘referral model’ for implementing the PtDA, which proposes that these tools are ‘adjuncts’ that support decision-making, when used ahead of visits or shortly afterwards.^31^ All patients (and their parents/carers) will have immediate access to the PtDA, regardless of whether they return their interview consent form. This is because we feel it would be unethical to withhold access to a resource that may be supportive and of benefit. Patients who wish to participate in the study will be instructed to contact the research team directly.

#### Data collection

While many measures can be used to evaluate the effectiveness of a PtDA, consultations with our PPI team and collaborators suggest that a purely qualitative approach is most appropriate given the young target age of our resource. In addition, many of the existing questionnaires are not written in a language that is suitable for use with this young age group. The aim of the interviews is to understand how the resource supports boys and young men to make reasoned FP treatment decisions and to explore its usability and acceptability with them, their parents/carers (where appropriate) and oncology and fertility service staff.

Semi-structured interviews with approximately 24 boys and young men (selected for demographic diversity) and 10 fertility and oncology staff will be carried out to gain a deeper insight into their experiences of using the PtDA. It is anticipated that some of the interviews with the participants will be dyadic or possibly triadic, that is, they may involve two or more family members depending on age, maturity and the wishes of the young person. The interviews will be carried out within 4 months of recruitment to the study, and after the patient’s first round of chemotherapy has been completed. Within this time frame, each patient will be given the opportunity to take part in the interview whenever is best for them.

Interviews will be conducted in an informal setting or online based on the patient’s wishes and facilitated by peer-led/paired interviews. Likely areas that the interviews will focus on include the resources’ usefulness for making decisions between treatment options, barriers to use in practice and whether or not the patients, parents/carers and HCPs found benefit from it.

#### Data analysis

The interviews will be recorded, transcribed verbatim and analysed using NVivo software. We will compare the one-to-one and dyadic interviews, informed by recent literature on the benefits and challenges of interviewing in pairs.^34^ Transcripts will be analysed using reflexive thematic analysis,^35^ an established approach that members of the team have used in previous research concerning male infertility.^16 36^ The analysis will be data-driven so that patient perspectives and language are prioritised with the final themes agreed following team data analysis sessions and discussions.

### Stage 4: production of final resource and completion of dissemination (months 34–36)

Stage 4 aims to produce the final version of the PtDA and disseminate findings from the study. Suggested revisions from the beta testing phase will be shared with the study team and expert steering group during a 1 day meeting for consultation and discussion. Based on our experience, it is likely that any modifications requested at this stage will be minor. The PtDA will then be sent to the design team to complete the final revisions, and a PDF version of the booklet will be uploaded to the CFM PtDA website. During the study, we will also apply for additional funding to create a theatre performance (in line with PPI suggestions and expertise) to disseminate our resource and findings at the study’s end.

### Ethics and dissemination

This study has been prospectively registered on the Research Registry (10273). Ethics approval has been obtained from Leeds Beckett University (141879) and the Health Research Authority (HRA) (REC committee reference: 24/WM/0141). The study will conform to the UK Framework for Health and Social Care Research and the British Psychological Society’s Human Research Ethics guidelines. The principle of ‘Gillick competence’ will be applied with regards to consent for research.

The multidisciplinary and collaborative nature of this proposal will enable us to disseminate the study and its milestones into the NHS and wider healthcare community through a variety of local and national channels. This will be achieved by:

- Sharing our findings and progress with a range of stakeholders, including cancer charities (eg, Children with Cancer UK) and other national organisations (eg, CCLG).
- Communicating the study via professional organisations which have special interest groups on FP, such as the British Fertility Society and the European Society for Human Reproduction and Embryology.
- Communicating the study via a dedicated project webpage, institutional media teams and other social media channels, for example, Twitter/X (@CFMyoungmen), Facebook, TikTok and Instagram.
- Circulating the clinical implications of the research via attendance at professional meetings, such as the CCLG Spring Meeting and Teenage and Young Adult Cancer Congress.
- Using standard routes, including conferences and open-access publications.
- Engaging with patients and the public through the development of a variety of study summary products, including audio-visual and short written pieces.
- Uploading the PtDA to the existing CFM website, ensuring it is freely available to boys and young men with cancer, their parents/carers and fertility and oncology HCPs.
- Preparing an application to IPDAS for inclusion in their A–Z PtDA inventory. Our female version achieved full marks by IPDAS as a resource to support women of reproductive age to make FP decisions; we hope to achieve the same standard with this new resource.
- Producing a set of dissemination ‘business cards’ that will include the links to the resource and sending these to cancer centres for use with their patients.
- Applying for further funding to create a theatre performance in collaboration with PPI expertise.

## Discussion

The primary goal of this study is to create and evaluate a novel, evidence-based PtDA tailored for boys and young male patients with cancer aged 11–25 years old to consider their FP options. It is anticipated that the development of this resource will provide empirical evidence about the effectiveness of a PtDA to support boys and young men to make complex decisions about FP in the context of a cancer diagnosis.

As far as we are aware, there are no existing FP PtDAs for boys and young men; members of the study team have successfully developed an equivalent PtDA for young women^32^ and it is hoped that this acts as a strong basis for the development of a similar, open-access PtDA for young men.

A strength of this research is that it is led by a strong and experienced team, working collaboratively with PPI stakeholders to ensure patient needs and wants are at the centre of this work. Another strength is that the PtDA will be delivered early into the cancer care pathway, providing clinical teams with an evidence-based resource that they can give to all boys and young men (and their parent/carers where relevant) diagnosed with cancer. It is anticipated that this will complement the discussions that cancer specialists have with young men and their families, as well as facilitate open conversations between boys and young men and their families, supporting them to make sensitive decisions collaboratively.

Methodological limitations of this research include the proposed sampling strategies in stages 2 and 3. Recruitment time may be limited due to the NHS/HRA approval process and time required to complete site set-up. Suboptimal recruitment has been reported in previous PtDA development studies, including our CFM programme of work for adult women.^32^ Reported barriers to our ‘patient referral model’ of recruitment include HCP hesitancy, including demanding workloads, prioritising cancer treatment decisions and delivery, and lack of confidence in the content and benefit of the PtDA. However, the small sample size should minimise HCP burden, and we will delegate tasks to oncology nurses to increase implementation of the PtDA.^32^ Recruitment rates will be monitored throughout the study, and we will introduce new strategies if required.

The data collected through this study will enable us to understand factors influencing the practical application and integration of the PtDA into clinical practice, advancing the understanding already gained through our young female project. As part of the evaluation process, healthcare teams involved in cancer care will distribute information about the study and the PtDA. We will gather feedback from these teams regarding the feasibility of integrating this approach into their care practices. In conclusion, this research aims to fill a critical gap by developing and evaluating an open-access PtDA tailored for boys and young men with cancer, with the ultimate goal of improving decision-making around FP, informed by empirical evidence and guided by patient-centred care principles.
